# Supplementary material for: Parental Decision Making Regarding COVID-19 Vaccines for Children under Age 5: Does Decision Self-Efficacy Play a Role?
Source: Vaccines (Basel). 2023 Feb 18;11(2):478. doi: 10.3390/vaccines11020478 (PMC9959902; doi:10.3390/vaccines11020478)
Supplement: Supplementary file 1 [file vaccines-11-00478-s001.zip › vaccines-2133168-supplementary.pdf]

Supplementary Material: Parental decision-making about COVID-19 vaccines for children under age 5: Does Decision Self-Efficacy play a role?

Vaccine Decision Self-Efficacy questions adapted from O'Connor et al.[1]

How confident are you that you can:

1. Get the information you need to make a decision about getting vaccines
2. Get the facts about the benefits of vaccines
3. Get the facts about the risks of vaccines
4. Understand available information about vaccines enough to be able to make an informed decision about vaccination
5. Ask your health care provider questions about vaccines without feeling dumb.
6. Express your concerns about vaccines to your health care provider.
7. Figure out the vaccination choices that best suit you personally
8. Ask for advice about getting vaccines
9. Handle unwanted pressure from others when making your choice about vaccination
10. Tell your healthcare provider what you think is right for you
11. Delay your decision if you feel you need more time to make decisions about vaccines

Response options: Not at all confident, somewhat confident, very confident

Reference

1. O'Connor, A.M.; Tugwell, P.; Wells, G.A.; Elmslie, T.; Jolly, E.; Hollingworth, G.; Mcpherson, R.; Drake, E.; Hopman, W.; Mackenzie, T. Randomized Trial of a Portable, Self-administered Decision Aid for Postmenopausal Women Considering Long-term Preventive Hormone Therapy. *Med. Decis. Mak.* **1998**, *18*, 295–303. https://doi.org/10.1177/0272989x9801800307.
